# Utility of smart watch in expediting diagnosis of cold drink-triggered atrial fibrillation: a case report

**DOI:** 10.1186/s12245-024-00716-z

**Published:** 2024-10-08

**Authors:** Grace V. Heringer, David R. Vinson

**Affiliations:** 1 Department of Neurobiology, Physiology and Behavior, University of California, Davis, California USA; 2 The Permanente Medical Group, Kaiser Permanente Northern California Division of Research, and the CREST Network, Pleasanton, California USA; 3 Department of Emergency Medicine, Kaiser Permanente Roseville Medical Center, 1600 Eureka Road, 95661 Roseville, California USA; 4 Department of Emergency Medicine, University of California Davis Medical Center, Sacramento, United States

**Keywords:** Atrial fibrillation, Paroxysmal tachycardia, Emergency medicine, Electrocardiography, Precipitating factors, Palpitations, Cold drink, Smart watch

## Abstract

**Background:**

Patients presenting to the emergency department with recent palpitations are a diagnostic challenge when the arrhythmia and its symptoms have resolved prior to arrival. Newer smart watch technology, adept at detecting atrial fibrillation, can assist in the diagnostic evaluation of transitory palpitations. We report a case of cold drink-triggered atrial fibrillation whose diagnosis would not have been possible without the assistance of the patient’s smart watch.

**Case presentation:**

A middle-aged man without cardiac history developed sudden rapid, irregular palpitations with shortness of breath while drinking a glass of cold juice. He activated his smart watch with 1-lead electrocardiography technology which detected rapid atrial fibrillation. He sought medical care, but while waiting, his symptoms—then 90 min in duration—spontaneously resolved. His initial diagnostic evaluation documented only sinus rhythm, as did several follow-up evaluations with cardiology the next several weeks. Had it not been for his smart watch, the etiology of his transitory palpitations would have remained undiagnosed. His physicians encouraged trigger avoidance. In the following months, he avoided rapid ingestion of cold drink, taking instead small sips. The atrial fibrillation has not recurred.

**Conclusions:**

The case illustrates the valuable contribution of smart watch technology in the diagnostic evaluation of patients with short-lived palpitations. The case also educates clinicians about cold drink and food as a trigger of paroxysmal atrial fibrillation. This trigger, like alcohol, can be modified. Cold drink trigger avoidance has been reported by patients to reduce atrial fibrillation recurrence and is a low-risk, cost-effective strategy that is often successful.

## Background

Palpitations—sensations of rapid, irregular, or forceful pulsations in the chest—are prevalent among patients presenting for emergency department (ED) care [1]. Approximately one-third of patients with palpitations in U.S. EDs have a cardiac etiology. This is why a 12-lead ECG coupled with continuous cardiac monitoring are essential to the ED diagnostic evaluation, especially knowing that some cardiac causes of palpitations can be life-threatening and readily treatable [1, 2]. Many arrhythmias, however, are transitory in nature, often self-resolving before ED presentation. When evading capture by ED monitoring, these elusive arrhythmias can prolong—and even frustrate—the diagnostic process. In this regard, recent improvements in smart watch technology can be a diagnostic game-changer [3]. Advanced wearable devices allow patients to contribute invaluable intel to the diagnostic process. Capturing short-lived arrhythmias before they go underground, patients and their wearables can greatly expedite diagnosis, work-up, and treatment.

One arrhythmic cause of palpitations commonly diagnosed in the ED is atrial fibrillation (AF) [1, 4–6]. Susceptible patients may develop paroxysmal AF immediately following ingestion of cold drink or food, a condition sometimes called Cold Drink Heart (a name styled after Holiday Heart, which is AF precipitated by alcohol binging) [7–9]. Because the anterior esophagus abuts the posterior heart, swallowed cold drink, or food, like ice cream or yogurt, can transiently chill the adjacent myocardium, which can precipitate AF through presumed vagal mediation [10]. Cold Drink Heart has not been well studied but has been reported in a series of case reports, dating back at least to 1985 [10–17]. It is thought to occur more commonly in younger patients without cardiac disease, though it can develop in older patients with structural heart disease [18]. Whatever the mechanism and epidemiology, identification of a modifiable lifestyle trigger like cold drink or food allows for trigger avoidance in pursuit of AF reduction, as the following case illustrates.

## Case presentation

**Fig. 1:**
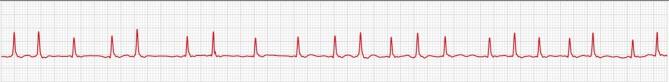
One-lead electrocardiogram from smart watch identifying atrial fibrillation shortly after symptom onset

A 43-year-old male presented to the urgent care with recent-onset palpitations and a concurrent smart watch (Apple Watch Ultra [Cupertino, CA]) interpretation of rapid AF. He had a history of gastroesophageal reflux disease, hyperlipidemia, and hypertension, all well-controlled with diet, daily exercise, rosuvastatin, and benazepril. That day, he had been working outside for an hour in the hot sun before going indoors to get a cold glass of vegetable juice from the refrigerator. He “chugged it” as fast as he could. Within a few seconds of the first big gulp, he developed racing, irregular palpitations with mild shortness of breath and slight dizziness. He had no chest pain nor limb complaints. He had no cardiac diagnoses but did report a similar episode of cold drink-induced palpitations 5 years earlier that self-resolved after 20 min. He had not sought medical care for the initial episode. Ten minutes after symptom onset with this second spell, he had his smart watch interpret his rhythm and pulse rate: (Fig. 1). The following sentences accompanied the tracing: “This ECG shows signs of AFib and a high heart rate. If this is an unexpected result, or your heart rate stays high, you should talk to your doctor soon.” Two subsequent readings over 30 min were similar. This information prompted a trip to urgent care.

**Fig. 2:**
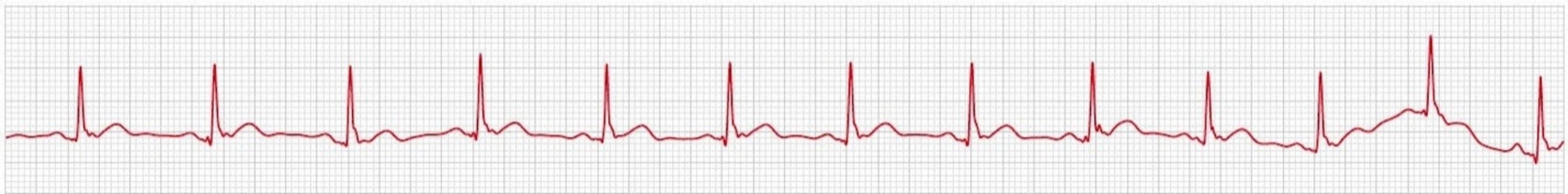
One-lead electrocardiogram from smart watch identifying normal sinus rhythm after spontaneous cardioversion

While waiting to be seen, the patient’s symptoms resolved, as did his AF, 90 min after it had begun. His first 12-lead ECG revealed sinus rhythm with a heart rate of 69 beats/minute, similar to a concurrent smart watch tracing (Fig. 2). The remainder of his vital signs were normal, as was his physical examination. He was transferred to a nearby ED for further evaluation. There, his temperature was 98.5 °F; blood pressure, 135/83 mmHg; pulse rate, 70 beats/minute; respiratory rate, 20 breaths/minute; and peripheral oxygen saturation, 99% on room air. A 12-lead ECG showed normal sinus rhythm with normal intervals and no ST or T-wave abnormalities. A portable chest radiograph was unremarkable. His laboratory values were normal, including complete blood count, basic metabolic panel, magnesium, phosphorus, D-dimer, and serial high-sensitivity troponins. His CHA_2_DS_2_VASc score was 1 (for hypertension), not meeting definitive criteria for anticoagulation [4, 5]. 

The treating emergency physician diagnosed him with paroxysmal AF triggered by cold drink. He was discharged home with instructions to avoid cold drink and to take 81 mg of aspirin daily until he saw a cardiologist the following week. The cardiologist ordered further studies: an echocardiogram identified no significant structural abnormalities and an exercise treadmill test was negative for ischemia. The cardiologist also encouraged trigger avoidance. The patient has since avoided rapid ingestion of cold drink, instead taking slow, small sips of cold beverages. He has had no further AF recurrences.

## Discussion

This case of smart watch detected cold drink-induced paroxysmal AF illustrates several practical lessons for clinicians who care for patients with palpitations. The first lesson is the valuable role direct-to-consumer smart watches can play in helping identify arrhythmic causes of palpitations. The arrhythmia most reliably detected by wearable smart devices—and the one most studied—is AF [3, 19, 20]. A recent prospective study of 5 commercially available smart devices tested their ability to detect and correctly identify AF in a real-world cohort of 201 cardiology patients [21]. 30% of patients were in AF at the time of testing. Devices commonly deemed the tracings inconclusive, the prevalence of which varied by device, ranging from 17 to 26%. Though the smart devices were unable to interpret inconclusive tracings, cardiologists were able to interpret nearly all of them. This demonstrates the need for physician confirmation or overread of smart device tracings, both those the device could and could not interpret. Nearly all tracings taken in the study were clinically useful in the AF diagnostic evaluation. Apart from inconclusive cases, these 5 smart devices performed equally well, with high sensitivity and specificity for AF [21]. Other studies have also required clinician arrhythmia confirmation, as in our case [22, 23]. 

The critical contribution to palpitation diagnostics made by smart watches is accentuated when the limitations of post-discharge monitoring are highlighted. Our patient had 2 palpitation episodes over 5 years and was unlikely to benefit from a short run of home monitoring. Outside the cardiology suite, generalists are advised to “…restrict a 24-hour Holter monitor to patients who have at least daily symptoms, a 48-hour Holter monitor to those with symptoms on most days, and a seven-day monitor to those with weekly symptoms, even if the 12-lead [ECG] is normal. Inappropriate use of short periods of ambulatory monitoring for infrequent symptoms is cumbersome for patients, delays the diagnosis, and is costly.” [24].

Lacking a tracing of the cold-induced rhythm 5 years prior limits us from assigning it a diagnosis of AF with certainty, as other etiologies are possible [19, 20]. AF, however, is the most probable etiology for that prior episode given his recent AF diagnosis.

The second lesson we can learn is that some cases of paroxysmal AF have modifiable ingestion triggers, in this case, cold drink, a condition sometimes called Cold Drink Heart [7, 8, 10, 16–18, 25]. Some patients are more susceptible to AF when cold trigger exposure occurs following exercise or strenuous exertion [16, 17]. Patients with cold drink or food-triggered AF have variable degrees of cold sensitivity. In some patients, AF is always or nearly always triggered with rapid ingestion of cold drink, which is not true for most Cold Drink Heart patients.

Helping patients make the causal connection between trigger and AF—or simply validating a connection patients have already drawn—allows clinicians to collaborate with their patients in devising a strategy for trigger reduction. Many clinicians, however, are unaware that cold drink and food can trigger AF and have been known to dismiss patient claims that cold drink precipitates their paroxysmal AF [7]. To address this, one large U.S. medical group included a cold drink prompt for physicians in their clinical decision support tool for the comprehensive management of AF in the ED [26]. The support tool reminded physicians to ask patients with intermittent AF if they had noticed that ingesting cold drink/food precipitated AF within seconds or minutes of ingestion. Along with the cold drink prompt, the support tool reminded physicians to inquire about alcohol ingestion, another modifiable AF trigger [9, 27–29]. 

Cold drink avoidance has been reported by Cold Drink Heart patients to reduce AF recurrence [7, 10, 18]. A study of patients with trigger-induced paroxysmal AF found trigger reduction decreased AF events [29]. The number of patients in this trial with triggers of cold drink or food was quite small (*n* = 5), preventing a meaningful analysis of this particular subcohort. The relative safety and low cost of a trigger reduction strategy lends itself to an informal *n* = 1 trial. If successful, trigger reduction may be a simple means of reducing the number of AF episodes, which may reduce AF-related symptoms, need for urgent medical care, and missed days of work.

In sum, we report a case of cold drink-triggered AF whose diagnosis was aided by an informative 1-lead ECG tracing captured by a smart watch. Clinicians caring for patients with transitory palpitations should inquire about cold drink triggers and use available smart watch tracings as diagnostic adjuncts.

## Data Availability

No datasets were generated or analysed during the current study.

## Abbreviations

AF: Atrial fibrillation
ED: Emergency department
ECG: Electrocardiogram
